# Krüppel-like Factor 4 and Glucocorticoid Receptor Cooperatively Transactivate the Bovine Alphaherpesvirus 1 (BoHV-1) Infected Cell Protein 0 (bICP0) Early Promoter

**DOI:** 10.3390/v18080884

**Published:** 2026-08-12

**Authors:** Hafez Sadeghi, Vanessa Claire Santos, Clinton Jones

**Affiliations:** Department of Veterinary Pathobiology, College of Veterinary Medicine, Oklahoma State University, Stillwater, OK 74078, USA; hafez.sadeghi@okstate.edu (H.S.); vanessaclaire.santos@okstate.edu (V.C.S.)

**Keywords:** bovine alphaherpesvirus 1 (BoHV-1), pioneer factors, glucocorticoid receptor (GR), Krüppel-like factor 4 (KLF4), infected cell protein 0 (bICP0) E-promoter

## Abstract

Bovine alphaherpesvirus 1 (BoHV-1) acute infection induces respiratory tract disorders and conjunctivitis and suppresses immune responses that may cause bacterial pneumonia. BoHV-1 infection establishes lifelong latency in sensory neurons in trigeminal ganglia (TG), the central nervous system, and certain cells in the pharyngeal tonsil. BoHV-1 is a chronic problem in the cattle industry because stress, including the synthetic corticosteroid dexamethasone, triggers reactivation from latency after an intravenous injection. The BoHV-1 immediate early transcription unit 1 (IEtu1) promoter drives expression of infected cell protein 0 (bICP0) and bICP4, two viral transcriptional regulators. Stress activates the glucocorticoid receptor (GR), and Krüppel-like factor 15 (KLF15) cooperatively transactivates the BoHV-1 IEtu1 promoter if both GR response elements (GREs) are intact. Since the bICP0 gene contains a separate early (E) promoter, we tested the hypothesis that GR+KLF family members transactivate the bICP0 E-promoter. GR+KLF4, both pioneer transcription factors, cooperatively stimulate bICP0 E-promoter activity in mouse neuroblastoma cells (Neuro-2A), and stimulate productive infection. Notably, the bICP0 E-promoter lacks GREs, suggesting that a novel mechanism triggers transactivation. CA motifs and C-rich Sp1 binding sites in bICP0 E-promoter sequences are crucial for transactivation and binding to GR and KLF4.

## 1. Introduction

Bovine herpesvirus 1 (BoHV-1) continues to be a persistent problem in the cattle industry, in part because it is a cofactor in bovine respiratory disease (BRD), a poly-microbial disease [1,2,3,4]. Furthermore, BRD is the most economically important disease affecting beef and dairy cattle because approximately 75% of morbidities and more than 50% of mortalities in feedlot cattle are caused by BRD. A commensal bacterium, *Mannheimia haemolytica* (*MH*), is normal flora in the upper respiratory tract of healthy ruminants, reviewed in [5,6,7,8]. This commensal relationship is disrupted following stress or co-infections. *MH* generally causes bronchopneumonia in BRD cases [8,9]. BoHV-1 infection generally causes upper respiratory tract disease and erodes mucosal surfaces of the upper respiratory tract [10,11]. Consequently, MH colonizes the lower respiratory tract, promoting interactions between the *MH* leukotoxin and bovine peripheral blood mononuclear cells, including neutrophils. Finally, BoHV-1 acute infection impairs immune responses, cell-mediated immunity, CD8+ T cell recognition of infected cells, and CD4+ T cell functions, reviewed in [12,13].

**Figure 1:**
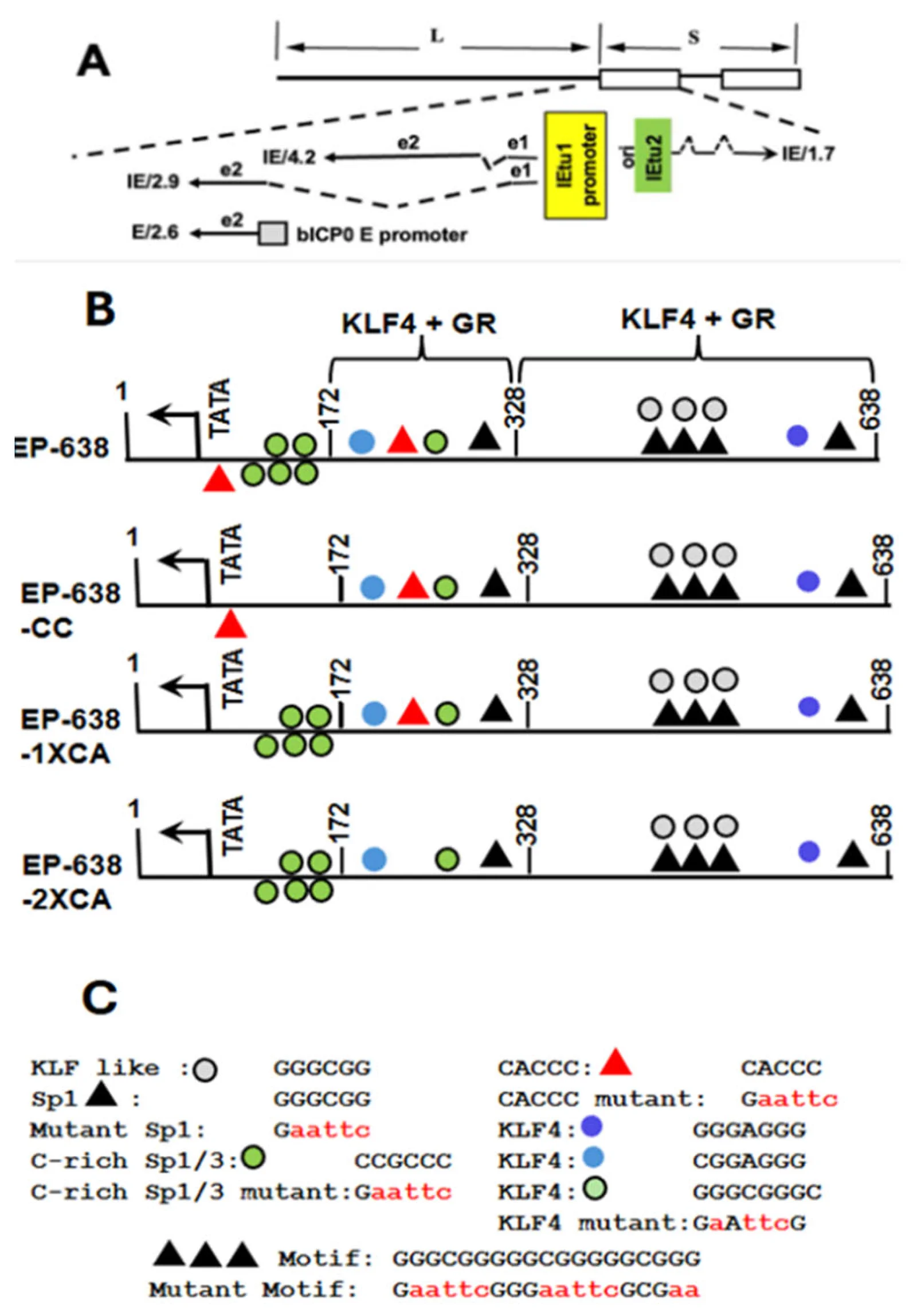
Schematic of BoHV-1 genome and immediate early transcription unit 1 (IEtu1). Panel (**A**): Schematic of BoHV-1 genome. Sequences in the unique long sequences is depicted as L, unique short sequences is depicted S, and open rectangles signify the BoHV-1 repeats. Alternatively spliced IE transcripts (IE/4.2 and IE/2.9) are translated into two transcriptional regulatory viral proteins (bICP4 and bICP0). The yellow rectangle is the EItu1 promoter that drives expression of two IE mRNAs (IE/4.2 and IE/2.9) during productive infection. IE/4.4 is translated into bICP4 and IE/2.9 is translated into bICP0. Expression of a novel bICP0 transcript (E/2.6) is driven by a separate bICP0 early promoter (bICP0 E-promoter) depicted by the grey rectangle. The other IE promoter (IEtu2; green rectangle) drives expression of the IE/1.7 transcript that is translated into the bICP22 protein. Solid lines in the transcripts signify exons (e1, e2, or e3) and dashed lines are introns. The origin of replication (ORI) is adjacent to the IEtu2 promoter. Panel (**B**): EP-638 is upstream of the luciferase vector (pGL3-Basic Vector (Promega, Madison, WI, USA)), as previously described [23]. The position of the TATA box, and arrow denote the position and direction of the bICP0 mRNA start site. Mutations in EP-638 sequences were synthesized by GenScript, and these mutants were inserted into the multiple cloning site of the pGL3-Basic Vector. Panel (**C**): Potential KLF4, Sp1, and/or Sp3 binding sites are identified by colored circles. These potential binding sites may be influenced by adjacent transcription factor binding sites and chromatin status. Mutations of denoted Sp1 or KLF4 binding sites were mutated by inserting an EcoRI restriction site (GAATTCC) in place of the respective mutations.

In addition to high levels of BoHV-1 virus production in mucosal epithelial cells, BoHV-1 also infects CD4+ T cells, which induces apoptosis and inflammation but not high levels of virus production. Like other alpha-herpesvirinae subfamily members, viral gene expression occurs in three phases: immediate early (IE), early (E), and subsequently late (L). In contrast to herpes simplex virus 1 (HSV-1) and HSV-2, IE transcription unit 1 (IEtu1) encodes two transcriptional regulatory proteins (bICP0 and bICP4), which stimulate productive infection (Figure 1A) [14,15,16,17]. A bICP0 E-promoter is also present in IEtu1, which drives high levels of bICP0 during productive infection [14,15,16,17]. The only other IE transcript encodes the bICP22 protein. A virion protein, VP16 and two cellular transcription factors (HCF-1 and Oct-1) form a complex that binds sequences in all IE promoters, culminating in IE promoter activation [17]. Early mRNA expression occurs in the absence of viral DNA replication and is generally translated into non-structural proteins that mediate viral DNA replication. The L mRNA expression generally requires viral DNA replication, and L proteins are part of the infectious virus particles.

Viral replication in the oral, nasal, or ocular cavity during acute infection culminates in production of infectious viruses that enter sensory neurons in trigeminal ganglia (TG) via synaptic connections, reviewed in [12,13]. Lytic cycle viral gene expression is limited and low levels of virus production occur in infected TG sensory neurons. Viral gene expression is extinguished in many infected neurons, and certain neurons survive infection. The surviving infected neurons contain viral DNA, and latency is established. The only viral RNA abundantly expressed in latently infected neurons encodes the latency-related (LR) gene products and ORF-E, which is upstream of the LR gene. A small non-coding RNA, encoded within LR gene sequences, impairs bICP0 expression [18]. Stress, as mimicked by the synthetic corticosteroid hormone dexamethasone (DEX), consistently induces BoHV-1 reactivation from latency, reviewed in [12,13]. Generally, stress activates the glucocorticoid receptor (GR), reviewed in [19]. GR homodimers bound to DEX enter the nucleus, remodel chromatin, and trigger transcription within minutes because de novo synthesis of proteins is not required.

Previous studies demonstrated that the IEtu1 promoter and bICP0 E-promoter are occupied by GR within 3 h after latently infected calves are treated with the synthetic corticosteroid (DEX) [20]. Notably, the IEtu1 promoter contains two consensus GR response elements (GREs) that are essential for GR-mediated transactivation [21], but the bICP0 E-promoter does not contain a consensus GRE. However, GR and Krüppel-like factor 4 (KLF4), both pioneer transcription factors, cooperatively transactivate the bICP0 E-promoter [22]. The focus of this study is to identify bICP0 sequences necessary for GR- and KLF4-mediated transactivation of the bICP0 E-promoter.

## 2. Materials and Methods

**Cell culture and transfection:** Murine neuroblastoma cells (Neuro-2A) [23] were purchased from ATCC (catalogue no; ATCC CCL-131; Manassas, VA, USA) and grown in minimal essential medium (MEM) supplemented with 10% fetal bovine serum (FBS). MEM also contained penicillin (10 U/mL) and streptomycin (100 µg/mL) (Sigma-Aldrich catalogue no. P7539; St. Louis, MO, USA). Neuro-2A cells (~6 × 10^5^) were seeded into 60 mm tissue culture dishes that contained MEM purchased from Corning (catalogue no 10370021; Corning, New York, NY, USA) with 10% FBS Atlas Biologicals (FR-0500-A; Fort Collins, CO, USA) 24 h before transfection. Neuro-2A cells were transfected with the denoted luciferase reporter plasmid constructs (0.5 µg plasmid DNA) and a plasmid containing a minimal herpesvirus thymidine kinase (TK) promoter that expresses *Renilla* luciferase expression (50 ng DNA). An empty expression vector (pcDNA3.1) was used to have equal plasmid levels in the transfection mixture. Neuro-2A cells were incubated in MEM that included 2% charcoal-stripped FBS purchased from Sigma-Aldrich (catalogue no. F6765; St Louis, MO, USA) after cells were transfected. Stripped FBS is passed through a column containing “activated” charcoal to remove hormones, cytokines, and certain growth factors. Certain Neuro-2A cultures were treated with water-soluble DEX (100 nM) (D2915; Sigma-Aldrich), as denoted.

**Plasmids:** Dr. Joseph Cidlowski, NIEHS, provided us with the murine GR-α expression vector, which is denoted as GR throughout the manuscript [19]. Dr. Jonathan Katz (University of Pennsylvania) provided us with the KLF4 expression plasmid. The KLF15 expression plasmid was obtained from Dr. Deborah Otteson (University of Houston). The STAT3 expression vector was purchased from Addgene (plasmid #8706; 490 Arsenal Way, Suite 100, Watertown, MA, USA). Construction of the BoHV-1 bICP0 E-promoter construct was previously described (EP-638) [22]. All mutant promoter constructs shown in iyr studies were synthesized by GenScript (Piscataway, NJ, USA). These bICP0 E-promoter constructs are in the pGL3-Basic Vector (Promega, Madison, WI, USA). Schematics of these constructs are depicted in included in this manuscript. Plasmid DNA was prepared from bacterial cultures by alkaline lysis and two rounds of cesium chloride ultracentrifugation.

**Dual-luciferase studies:** Forty-eight hours after transfection, cells were collected, and protein extracts and the levels of luciferase was measured using a dual-luciferase assay kit purchased from Promega (E1910; Madison, WI, USA). Luminescence was measured by using a GloMax 20/20 luminometer (E5331; Promega).

**Chromatin immunoprecipitation studies (ChIP):** ChIP studies were performed as previously described [20,21,22]. Neuro-2A cells were grown in 100 mm dishes until ~80% confluency. Cells were transfected with the designated wild-type (wt) EP-638 or the designated mutant constructs (1.5 μg DNA) and the plasmid expressing KLF4 (1.5 μg DNA) and/or the GR expression construct (3 μg DNA) using Lipofectamine 3000 (Invitrogen; L3000015; Carlsbad, CA, USA), as described by the manufacturer’s instructions. After transfection, MEM with 2% stripped FBS was added to cultures. Two days post-transfection, Neuro-2A cells were cross-linked with paraformaldehyde and ChIP studies performed, as previously described. Cells were immunoprecipitated and incubated with a GR antibody (Cell Signaling, Cat# 3660S; Boston, MA, USA), KLF4 antibody (Abcam; Cat# ab106629; Cambridge, UK) or non-specific isotype control rabbit IgG (Abcam; Cat# ab171870; Cambridge, UK). DNA was purified using phenol–chloroform–isoamyl and amplified by PCR primers specific to the pGL4.24 plasmid [CGGAACGGAAGCGGAAAC (F) and ACAGTACCGGATTGCCAAG (R)]. Following agarose gel electrophoresis, DNA bands were quantified using Image Lab software (version 6.1 for Mac computers) and presented as % of the input sample.

**Figure 2:**
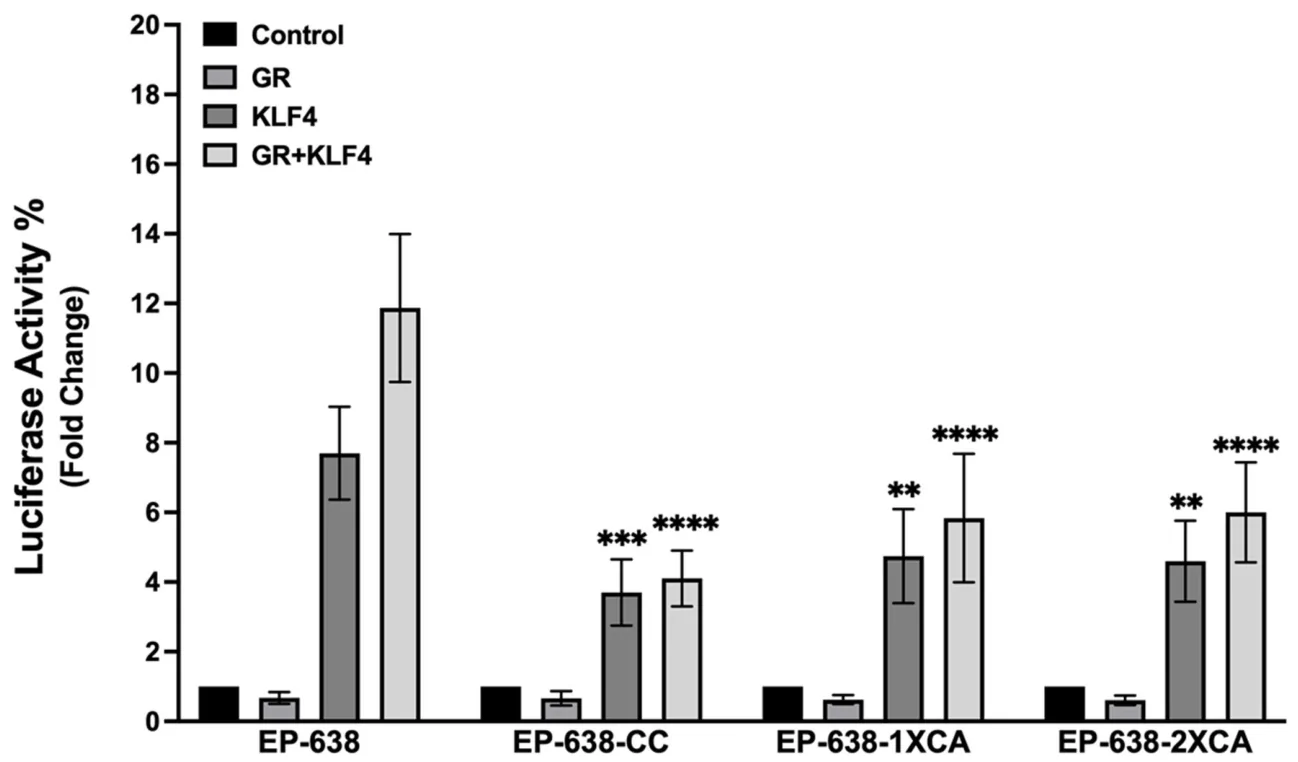
Mutating C-rich Sp1 binding sites impair transactivation of bICP0 E-promoter activation by GR and KLF4. Neuro-2A cells were co-transfected with the bICP0 E-promoter (EP-638 promoter; 0.5 µg DNA), or mutant constructs (EP-638-CC, EP-638-1XCA, EP-638-2XCA) along with plasmids that express GR and/or KLF4 proteins, as denoted. Cultures were treated with 2% stripped FBS after transfection. At 48 h after transfection, cells were harvested and protein lysate subjected to a dual-luciferase assay. Promoter activity levels in the sample containing wt EP-638 co-transfected with only an empty vector were normalized to a value of 1, and fold activation for other samples are presented. Statistical significance is defined as ** *p* < 0.01, *** *p* < 0.001, and **** *p* < 0.0001 compared with EP-638.

**Statistical analysis:** All statistical analyses were performed using GraphPad Prism Version 11.0.2. The results shown in all figures are presented as the mean ± standard error of the mean (SEM) from three independent experiments. The data were analyzed using two-way ANOVA followed by Tukey’s multiple-comparison test. In Figure 2, statistical significance is defined as ** *p* < 0.01, *** *p* < 0.001, and **** *p* < 0.0001 compared with EP-638. In Figure 4, statistical significance is defined as **** *p* < 0.0001 compared with wt EP-638, and a hash (#) denotes the significant differences between indicated groups (#, *p* < 0.0001). Statistical significance between indicated groups is defined as **** *p* < 0.0001, and ns indicates no statistically significant difference.

## 3. Results

### 3.1. C-Rich Sp1/Sp3 Consensus Sequences Within the bICP0 E-Promoter Are Important for GR- and KLF4-Mediated Transactivation

GR and Krüppel-like factor 4 (KLF4) are pioneer transcription factors [24,25,26,27] that cooperatively activate the bICP0 E- promoter [22]. The GC-rich (~80%) bICP0 E-promoter contains numerous potential KLF and Sp1 binding sites, plus two half-GREs; however, mutating the ½ GREs did not impair GR-mediated transactivation. For these studies, we utilized a bICP0 E-promoter construct denoted as EP-638, which contains two separate Sp1 sites, a KLF4 site, and a motif that contains three overlapping Sp1 binding sites (GGGCGG/GGGCGG/GGGCGG) (Figure 1B,C). The triple Sp1 site is important for GR and KLF4 transactivation [22]. Mouse neuroblastoma (Neuro-2A) cells were used for these studies because they have neuronal-like properties [23] and are readily transfected, whereas bovine cells in general are difficult to transfect.

As previously demonstrated [22], KLF4 alone transactivated wt EP-638 promoter activity approximately 8-fold (Figure 2). When Neuro-2A cells were co-transfected with GR and KLF4 expression plasmids, EP-638 promoter activity was stimulated approximately 12-fold, significantly higher than by the KLF4 or GR expression plasmids alone and was used in the transfection (Figure 2). GR can be phosphorylated by several protein kinases in the cytoplasm, consequently, GR enters the nucleus via binding to a GRE or can interact with other transcription factors, which are tethered to certain promoters. This is referred to as ligand-independent mediated transcriptional activation and increased levels of cortisol do not occur [28]. Addition of DEX does not increase promoter activity, which is consistent with previous studies that concluded GR transactivates bICP0 E-promoter activity via a ligand-independent mechanism [22].

The bICP0 E-promoter contains several C-rich Sp1/Sp3 consensus sequences upstream of the TATA box and are 3′ relative to position -172 (Figure 1B). There are also five KLF4 or KLF4-like motifs in EP-638, and two CACCC motifs. To test whether these motifs are important for GR- and KLF4-mediated transactivation of EP-638 promoter activity, additional mutant promoter constructs were constructed. For example, KLF4-mediated transactivation of EP-638-CC was significantly lower when compared to the wt EP-638 construct. Furthermore, the EP-638-CC promoter activity was significantly lower than the wt EP-638 construct when Neuro-2A cells were co-transfected with GR and KLF4, and promoter activity measured (Figure 2).

**Figure 3:**
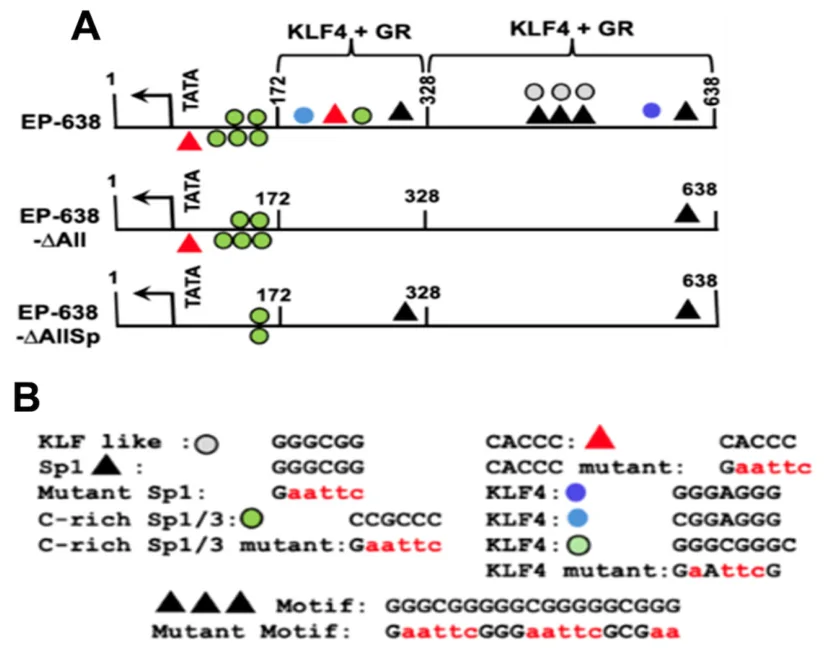
Schematic of bICP0 E-promoter and additional mutants. Panel (**A**): Schematic of additional bICP0 E-promoter constructs used for this study. EP-638 has been previously described [22]. The TATA box and arrow denote the position and direction of the mRNA start site. Mutations in the respective EP-638-∆All and EP-638-∆AllSp were synthesized by GenScript, and these respective fragments were inserted as a SacI-HindIII fragment into the multiple cloning site of the pGL3-Basic Vector, which drives expression of the luciferase reporter gene. Panel (**B**): Locations of key transcription factor binding sites are the same as those depicted in Figure 2. Mutations of the denoted Sp1 or KLF4 binding sites were disrupted by replacing the sequence with an EcoRI restriction site (GAATTCC).

The EP-638-1xCA construct contains a CACCC mutation adjacent to the TATA (Figure 1B). The promoter activity of EP-638-1XCA was significantly less than the wt EP-638 promoter activity when Neuro-2 cells were co-transfected with KLF4 or KLF4 plus GR (Figure 2). Moreover, EP-638-2XCA contains a CACCC mutation adjacent to the TATA box and between 172 and 328 bp regions of EP-638 (Figure 1B). When Neuro-2 cells were co-transfected with GR and KLF4, the EP-638-2XCA promoter exhibited significantly lower activity than the wt EP-638 construct (Figure 3). These studies revealed that mutating C-rich Sp1/Sp3 binding sites and/or the CACCC motif near the TATA box and between the TATA box to 172 and 328 bp regions of EP-638 were important. Interestingly, these mutated promoter constructs were also decreased by GR+KLF4 when compared to the wt EP-638.

### 3.2. CACCC Consensus Sequences Adjacent to the TATA Box and C-Rich Sp1/3 Sequences Were Crucial for GR- and KLF4-Mediated Transactivation

**Figure 4:**
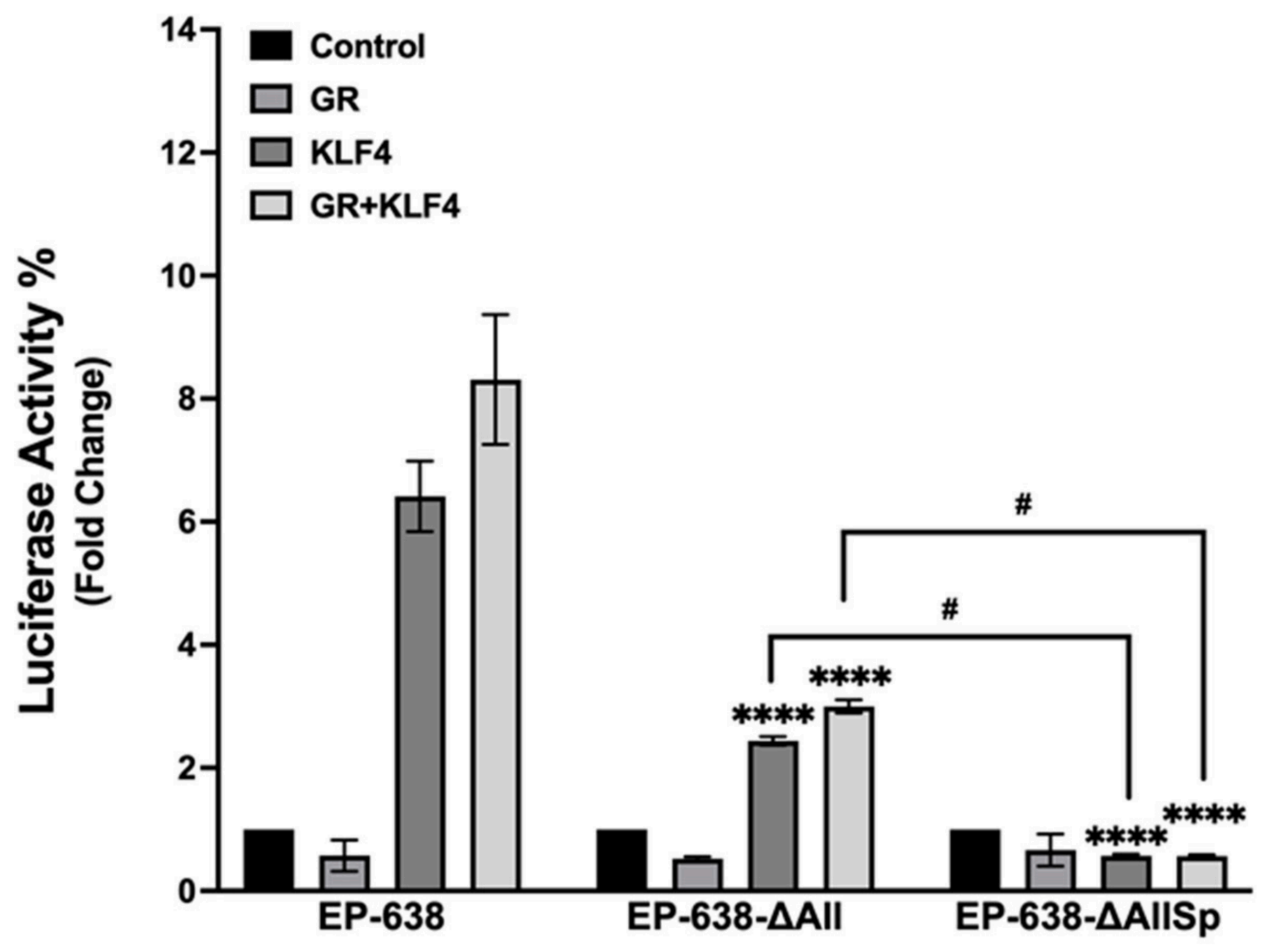
GR and KLF4 synergistically transactivate the bICP0 E-promoter. Neuro-2A cells were co-transfected with EP-638 promoter (0.5 µg DNA) or mutant constructs (EP638-All and EP638-AllSp) along with plasmids that express GR and/or KLF4 proteins. The designated cultures were treated with 2% stripped FBS. At 48 h after transfection, cells were harvested and protein lysate subjected to a dual-luciferase assay. Promoter activity levels in the sample containing wt EP-638 co-transfected with only an empty vector were normalized to a value of 1, and fold activation for other samples is presented. Statistical significance is defined as **** *p* < 0.0001 compared with wt EP-638, and a hash (#) denotes the significant differences between indicated groups (#, *p* < 0.0001).

The bICP0 E-promoter and two additional mutants (EP638-All and EP638-AllSp) (Figure 3) were compared to determine whether GR, KLF4, or GR-KLF4 contained differences in promoter activity in Neuro-2A cells. Consequently, we tested whether GC-rich Sp1 binding sites and KLF4 binding sites were important for GR- and KLF4-mediated transactivation of the bICP0 E-promoter. In contrast to EP-638-∆All, EP-638-∆AllSp contains a CACCC mutation adjacent to the TATA box and lacks three C-rich Sp1/3 sites but contains the G-rich Sp1 binding site near position 328. The EP-638-∆AllSp mutant exhibited significantly reduced KLF4-mediated transactivation relative to the wt EP-638 and EP-638-∆All (Figure 3). In Neuro-2 cells co-transfected with GR and KLF4, the EP-638-∆AllSp promoter showed significantly lower activity than wt EP-638 and EP-638-∆All exhibited near-basal promoter activity (Figure 4). These findings revealed that C-rich Sp1 binding sites and/or a CACCC motif near the TATA box were necessary for cooperative transcriptional activation of the EP-638 promoter by GR and KLF4.

### 3.3. STAT3 Does Not Cooperate with GR and KLF4 to Transactivate the bICP0 E-Promoter or IEtu1 Promoter

**Figure 5:**
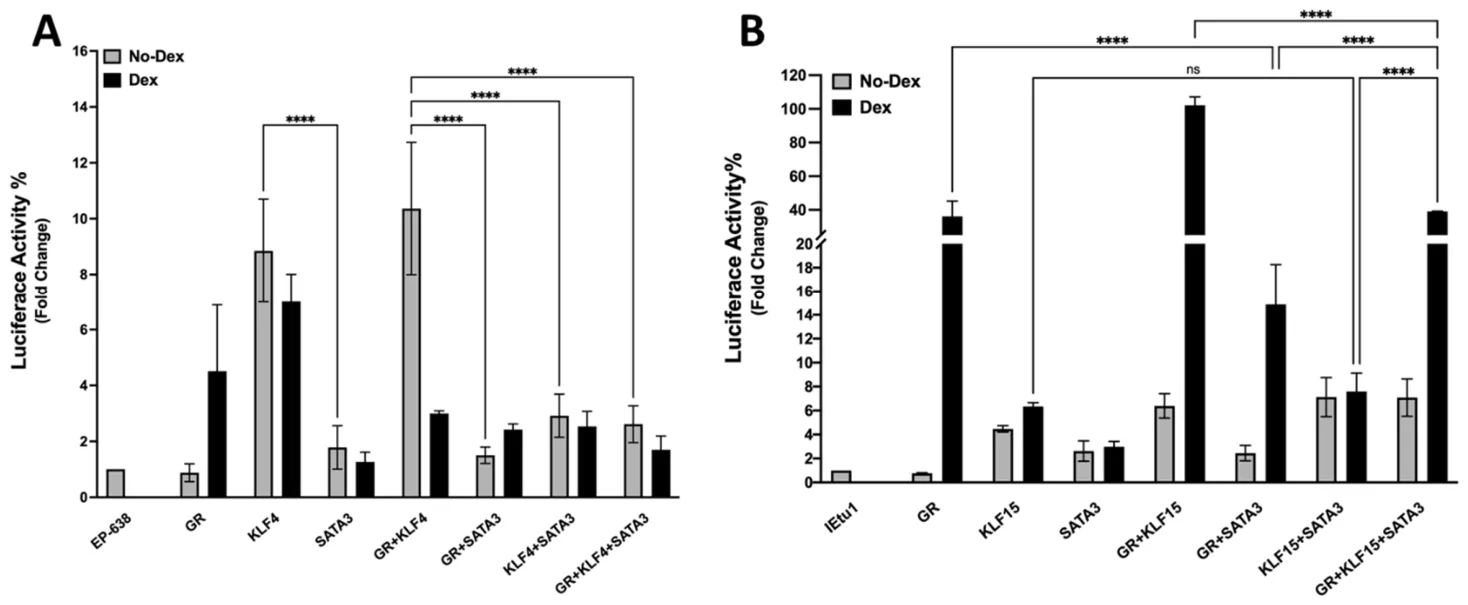
Effects STAT3 has on GR- and KLF4-mediated transactivation of the bICP0 E and IEtu1 promoters. Panel (**A**): Neuro-2A cells were co-transfected with EP-638 promoter construct (0.5 ug DNA) along with plasmids that express GR and/or KLF4 and signal transducer and activator of transcription 3 (STAT3) proteins. Certain cultures were treated with 2% stripped FBS. At 48 h after transfection, cells were harvested, a protein lysate was prepared and then subjected to a dual-luciferase assay. The wt EP-638 construct co-transfected with just an empty vector was normalized to a value of 1, and fold activation is presented for the other samples. Panel (**B**): Neuro-2A cells were co-transfected with the IEtu1 construct (0.5 ug DNA) along with plasmids that express GR and/or KLF4 and signal transducer and activator of transcription 3 (STAT3) proteins. Cultures were treated and promoter activity measured, as described in Panel (**A**). Statistical significance between indicated groups is defined as **** *p* < 0.0001, and ns indicates no statistically significant difference.

The rationale for examining whether the signal transducer and activator of transcription 3 (STAT3) transactivates the bICP0 E-promoter is because pseudorabies virus (PRV) reactivation from latency and gene expression is activated by STAT3 [29]. STAT3 is a transcription factor that can be a co-activator or inhibitor of GR-mediated transcription depending on the promoter, signaling environment, and availability of transcriptional cofactors. Notably, GR and STAT3 bind to the same genomic regulatory regions. GR tethering to DNA-bound STAT3 results in transcriptional repression, whereas STAT3 tethering to GR results in synergism [30]. Additional studies tested whether STAT3 cooperated with GR and KLF4 to transactivate the bICP0 E-promoter. As confirmed by previous studies, KLF4 and GR plus KLF4 transactivated the EP-638 promoter by approximately 8-fold and 11-fold, respectively, while DEX treatment reduced the promoter activity (Figure 5A). Interestingly, EP-638 promoter activity was not affected significantly by STAT3 and DEX treatment. In contrast to GR and KLF4, neither KLF4 and STAT3 nor GR and STAT3 enhanced EP-638 promoter activity. Furthermore, GR, KLF4, and STAT3 reduced EP-638 promoter activity by approximately 2-fold, which was significantly lower than the effects GR and KLF4 had on EP-638 promoter activity.

The IEtu1 promoter is synergistically transactivated by GR and KLF15; a feed-forward transcription loop [31]. Hence, we tested whether STAT3 cooperates with GR and KLF15 to transactivate the IEtu1 promoter. As expected, IEtu1 promoter activity was stimulated approximately 40-fold by GR and DEX treatment, approximately 6-fold by KLF15 and DEX treatment, and more than 100-fold by GR, KLF15, and DEX treatment (Figure 5B). Regardless of DEX treatment, STAT3 stimulated the IEtu1 promoter only 3-fold, however, which was significantly lower than IEtu1 transfected with GR in the presence of DEX. IEtu1 promoter activity was significantly reduced when GR and STAT3 were co-transfected when compared with GR and KLF15 following DEX treatment. IEtu1 promoter activity was significantly reduced when Neuro-2A cells were co-transfected with KLF15 and STAT3, compared to GR, KLF15, and cultures treated with DEX. In contrast to GR, KLF15, and DEX treatment, co-transfection of Neuro-2A cells with GR, KLF15, and STAT3 significantly reduced IEtu1 promoter activity to around 40-fold (Figure 5B). These results revealed STAT3 did not cooperate with GR to enhance the IEtu1 promoter activity. Furthermore, STAT3 significantly reduced GR- and KLF15-mediated activation of the IEtu1 promoter. In summary, these studies demonstrated that STAT3 does not have a dramatic effect on the bICP0 E-promoter (EP-638) or the IEtu1 promoter.

### 3.4. GR and KLF4 Occupy bICP0 E-Promoter Sequences in Transfected Cells

**Figure 6:**
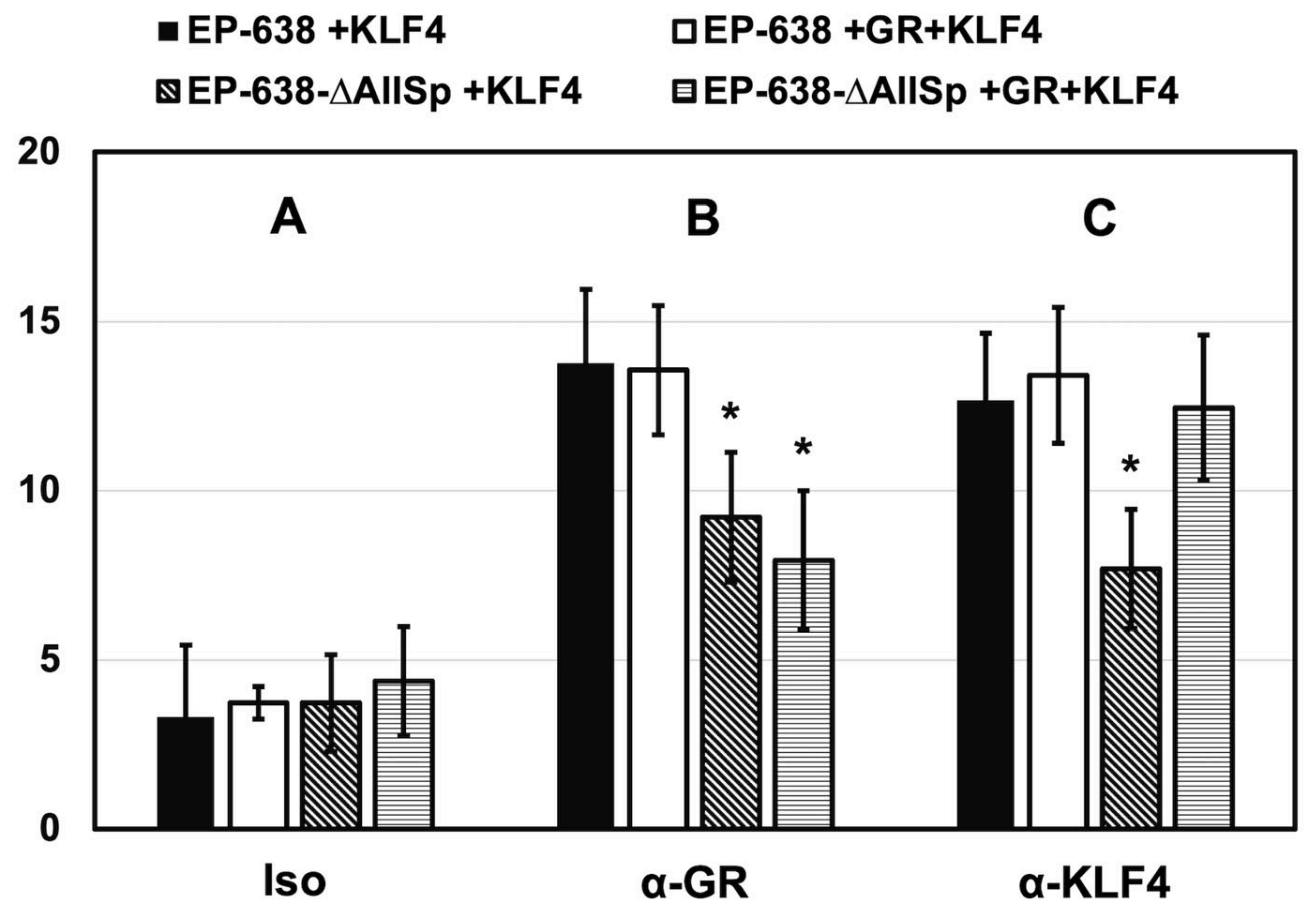
Association of GR and KLF4 with the EP-638 constructs in Neuro-2A cells. Neuro-2A cells were transfected with the wt EP-638 or mutant EP-638-∆AllSp promoter constructs, and KLF4 alone, GR expression plasmid alone, or GR and KLF4. Specific antibodies directed against GR (α-GR), KLF4 (α-KLF4), or a nonspecific IgG isotype control (Iso) were used for ChIP studies. Immunoprecipitated DNA was amplified by PCR and subsequently analyzed (Image Lab software). The data presented are a percentage of the input sample using the nonspecific IgG isotype control (Panel (**A**)) that is compared to the GR antibody (Panel (**B**)) or KLF4 antibody (Panel (**C**)). Occupancy of the bICP0 E-promoter by GR and KLF4 was significantly higher when the GR or KLF4 antibody was used for immunoprecipitation when compared to the isotype antibody, which is denoted by asterisks (*, *p*-value < 0.05). Statistics were performed using Student’s *t* test. These results represent three independent experiments.

To determine whether the Sp1 binding sites influence GR and KLF4 occupancy of the EP638 promoter, Neuro-2A cells were transfected with the wt bICP0 E-promoter construct (EP638) or the ΔAllSp mutant promoter construct with KLF4 alone or with KLF4 and GR. Chromatin immunoprecipitation (ChIP) studies were performed using a GR-specific antibody, KLF4-specific antibody, or an isotype antibody, as a negative control. Significant differences were not observed among groups immunoprecipitated with the isotype control antibody (Figure 6). ChIP analysis using a GR antibody revealed that GR occupancy of the wt EP638 promoter construct was similar in cells co-transfected with KLF4 alone or KLF4+GR. Conversely, GR occupancy of the EP-638-ΔAllSp mutant promoter was significantly reduced relative to the wt EP-638 promoter in cells co-transfected with KLF4 alone or KLF4+GR. Interestingly, ChIP analysis using a KLF4 antibody demonstrated KLF4 occupancy of the wt EP-638 promoter was not significantly altered by co-transfection with GR. KLF4 occupancy of the EP-638-ΔAllSp mutant promoter was significantly reduced compared to the EP-638 promoter in cells transfected with KLF4 alone. Interestingly, co-transfection of GR and KLF4 partially restored occupancy of the EP-638-∆AllSp promoter to levels of KLF4 comparable to the wt EP-638 promoter. These findings revealed that mutant sequences in the EP-638-∆AllSp construct contributed to efficient recruitment of both GR and KLF4 to the wt EP-638 promoter, whereas GR expression did not significantly alter promoter occupancy of either transcription factor.

## 4. Discussion

In contrast to BoHV-1, HSV-1 and HSV-2 contain distinct and separate ICP0 and ICP4 IE promoters. Our studies suggest this novel organization of the IEtu1 locus has the potential to efficiently reactivate from latency and enhance viral spread to numerous cell types during acute infection. For example, the bICP0 and VP16 proteins are detected prior to bICP4 in TG neurons during early stages of DEX-induced reactivation from latency [32]. This observation suggests the bICP0 E-promoter, not the IEtu1 promoter, drives bICP0 expression during early stages of reactivation from latency. Consistent with this observation, higher numbers of KLF4+ TG neurons are detected during DEX-induced reactivation from latency relative to latently infected calves and uninfected calves [33]. Like other pioneer transcription factors, KLF4 binds certain target sites in silent chromatin and directly or indirectly interacts with GR and other transcriptional coactivators. These steps promote remodeling of silent chromatin and triggering of transcription [24,25,26,27,34]. GR also exhibits pioneer factor functions because it binds a subset of GREs in silent chromatin that induces a nuclease-hypersensitive site that induces transcriptional activation [34,35]. Since herpesvirus genomes are chromatinized during latency, we predict pioneer factors are important for driving early stages of BoHV-1 reactivation from latency. In contrast to reactivation from latency in TG neurons, nearly all DEX-induced viral transcripts in pharyngeal non-neuronal cells of latently infected calves map to the bICP4 gene within 30 min after DEX treatment to induce reactivation from latency [36]. bICP4 and bICP0 RNA expression is detected in pharyngeal non-neuronal cells 90 min after DEX treatment, which supports the concept that the IEtu1 promoter preferentially drives bICP4 expression during early stages of reactivation from latency. It is also possible that alternative splicing of the IEtu1 primary transcript favors bICP4 expression. Within 3 h after DEX treatment, all viral transcripts are expressed. Thus, the IEtu1 promoter and bICP0 E-promoter are predicted to maximize lytic cycle viral gene expression in TG neurons versus non-neuronal cells in pharyngeal tonsil undergoing reactivation from latency. Furthermore, we predict that during acute infection, the IEtu1 promoter and bICP0 E-promoter drive productive infection in a cell-specific manner. A BoHV-1 mutant that contains a deletion of the TATA box in the bICP0 E-promoter exhibits similar virus titers in cultured bovine kidney cells but plaques are smaller (Jones, unpublished studies). Furthermore, this mutant does not cause severe disease in calves during acute infection and virus shedding is reduced relative to the rescued virus from nasal and ocular surfaces after DEX was used to induce reactivation from latency.

During latency, BoHV-1 lytic cycle regulatory genes are not detected in TG of calves using immunohistochemistry studies [32] and in situ hybridization [37]. Hence, cellular transcription factors must initially trigger viral gene expression during the transition from latency to reactivation from latency. The ability of GR+KLF4 to stimulate the bICP0 promoter in a ligand-independent manner may maintain viral gene expression long enough to activate lytic cycle genes and produce infectious virus in a small subset of latent neurons. Notably, there is no functional GRE in the bICP0 E-promoter, which suggests that increased cortisol is not always crucial for reactivation from latency. Enhancer elements within the bICP0 E-promoter are important for GR+KLF4-mediated transactivation downstream of the ICP4 IE4.2 mRNA [20,21,22], which may activate the IEtu1 promoter. Understanding the effects GR+KLF4 have on stimulating viral gene expression and where these pioneer transcription factors bind in the context of the entire genome will provide insight into how GR and KLF4 trigger viral gene expression following stressful stimuli.

The finding that GR and STAT3 had no effect on the bICP0 E-promoter and IEtu1 promoter was unexpected because STAT3 was reported to play an important role during pseudorabies virus (PRV) reactivation from latency in neuronal cells or in swine [32]. DEX treatment of calves latently infected with BoHV-1 consistently induces reactivation from latency, reviewed in [12,13]. Consistent with BoHV-1, 29 of 31 swine latently infected with PRV reactivated from latency after DEX treatment [38]. The BoHV-1 IEtu1 promoters (bICP0 E-promoter and IEtu1) are transactivated by GR, DEX, and certain stress-induced cellular transcription factors in several cell types, including Neuro-2A, Vero, and NIH3T3 cells [12,13,14]. These stress-induced cellular transcription factors were identified in TG during early stages of reactivation from 1 and 3 h after DEX treatment of latently infected calves [32,33]. In contrast to BoHV-1, the only immediate mRNA is PRV IE180 that encodes an ICP4-like protein [39]. Notably, the PRV IE180 promoter contains GREs, and is transactivated by GR and DEX treatment in Neuro-2A cells. However, PRV IE180 promoter is not transactivated by GR and DEX in a porcine kidney epithelial cell line (PK-15). Furthermore, PRV replication in PK-15 and Vero cells was reduced when treated with DEX. Despite stress and GR activation triggers for PRV and BoHV-1 reactivation from latency, several findings have revealed that virus-specific factors and species-specific factors appear to play important roles during early stages of reactivation from latency. We previously identified several cellular transcription factors and signaling pathways that are induced or repressed during DEX-induced BoHV-1 reactivation from latency in calves [33,36]. It would be interesting to compare cellular transcription factors and cell signaling pathways that are expressed during DEX-induced PRV reactivation from latency in latently infected swine. This would provide new insights into how two neurotropic alpha-herpesviruses trigger reactivation from latency in the natural host.

The bICP0 E-promoter lacks a consensus GRE whereas the two GREs in the IEtu1 promoter are essential for GR-mediated transactivation. Notably, Sp1- and KLF-like consensus sequences are present throughout the bICP0 E-promoter (Figure 1B,C). These studies focused on GC-rich Sp1 binding sites and KLF-like motifs present in the 5′ region. Although these studies revealed that they are important, this study provides evidence that C-rich Sp1 and CACCC motifs are also important for GR- and KLF4-mediated transactivation. Furthermore, these studies revealed that mutating Sp1- and KLF-like sequences proximal to the TATA box had a bigger effect than upstream motifs. For example, the EP638-∆All and EP638-AllSp were essentially not transactivated by GR and KLF4. In conclusion, these studies confirmed and expanded our understanding of how GR and KLF4 cooperatively transactivate the bICP0 E-promoter.

## 5. Conclusions

Since bICP0 is expressed prior to bICP4 during early stages of reactivation from latency in TG neurons, we predict bICP0 E-promoter activity drives bICP0 expression. Furthermore, pioneer transcription factors, including GR and Krüppel-like factor 4 (KLF4), are candidates that can remodel and activate bICP0 E-promoter activity during initial stages of reactivation from latency.

## Data Availability

In accordance with MDPI’s data policies, the data presented in this study are available upon request from the corresponding author. Raw data supporting the findings of this research will be made available in compliance with the journal’s guidelines and ethical standards for data sharing.
